# Molecular characterization of *Rhodococcus equi* isolates in equines

**DOI:** 10.14202/vetworld.2017.6-10

**Published:** 2017-01-05

**Authors:** Rabyia Javed, A. K. Taku, R. K. Sharma, Gulzaar Ahmed Badroo

**Affiliations:** Department of Microbiology, Faculty of Veterinary Sciences & Animal Husbandry, R.S. Pura, Sher-E-Kashmir University of Agricultural Sciences and Technology, Jammu, Jammu and Kashmir, India

**Keywords:** *16S rRNA*, polymerase chain reaction, *Rhodococcus equi*

## Abstract

**Aim:**

The aim was to determine the occurrence of *Rhodococcus equi* in equines and their environment in Jammu (R.S. Pura, Katra), molecular characterization and to determine the antibiotic resistance pattern of *R. equi*.

**Materials and Methods:**

A total of 96 nasopharyngeal swab samples were collected from equines. The organism was isolated on Columbia nalidixic acid agar containing 5% sheep blood as well as on sheep blood agar and was later confirmed by cultural characteristics and biochemical tests. Molecular detection of *R. equi* isolates was done by *16S rRNA* gene amplification followed by virulence associated protein A (*Vap A*) gene amplification. Antibiogram was performed against five antibiotics, *viz*., amoxicillin, penicillin G, streptomycin, rifampicin, and methicillin.

**Results:**

During the study, 9 *R. equi* isolates were identified on the basis of cultural and biochemical tests. In the polymerase chain reaction based detection, 3 among the 9 rhodococcal isolates were positive for species-specific *16S rRNA* gene and revealed amplicon of 450 bp for confirmation of *16S rRNA* gene. None of the sample was found positive for *Vap A* gene. In antibiogram, *R. equi* isolates were found sensitive for amoxicillin, while some isolates were also found resistant to the most conventional antibiotic penicillin G.

**Conclusion:**

From this study, it was concluded that *R. equi* infection is prevalent in equines in Jammu region of India and the indiscriminate use of the antibiotics is leading toward the development of resistant strains of *R. equi*.

## Introduction

For over 80 years, *Rhodococcus* *equi* has been recognized as a pulmonary pathogen of horses. The infection can spread from the lungs to other organs and joints when granulomatous foci in the lung open up, and infection of the gut lining causes diarrhea with an ulcerative enteritis and mucosal invasion of *R. equi* which is frequently observed in chronic disease. Immune complex deposition can cause polysynovitis which contribute to the development of uveitis, anemia or thrombocytopenia in infected foals. Occasionally, osteomyelitis and arthritis are also observed [1]. *R. equi* is Gram-positive, aerobic, nonmotile, nonsporulating, and metabolically diverse bacteria. The genus *Rhodococcus* (red pigmented cocci) belong to the phylogenetic group described as nocardia form actinomycetes. The infection causes subacute or chronic abscess or suppurative bronchopneumonia, ulcerative lymphangitis, enteritis, and causes zoonotic infection in foals aged 1-4 months [2,3].

Although infections can occur in healthy adult horses but are more common and severe in foals due to their immune compromised immunity. It has been found that only a small proportion of all *R. equi* in soil are able to cause the infection and only *R. equi* carrying virulence plasmids can cause disease in foals [4].

In some strains of *R. equi*, the presence of a plasmid encoding a 15-17 kDa protein called virulence associated protein A (*Vap A*) is known to be responsible for virulence [5]. In 85% of the cases, presence of *Vap A* virulence plasmid has been associated with *R. equi* infection in foals for the last couple of decades [6]. Experiments with the presence of the *Vap A* – expressing plasmid in *R. equi* showed an increase in the percentage of killed macrophages in a standard assay using trypan blue by roughly 20-70% in comparison with its cured equivalent strain.

The present investigation was undertaken to study the molecular diagnosis of *R. equi* including the therapeutics based on antimicrobial sensitivity tests.

## Materials and Methods

### Ethical approval

The approval from the Institutional Animal Ethics Committee to carry out this study was not required as no invasive technique was used. Nasal swab samples were being collected from clinically affected animals and healthy animals for this study as per standard collection procedure.

### Sample collection

Total 96 nasopharyngeal Swabs samples (both clinically affected and apparently healthy animals) were collected from equines and immediately transported to the laboratory on ice for further processing. The laboratory work was conducted in the Division of Veterinary Microbiology and Immunology, Faculty of Veterinary Sciences and Animal Husbandry, Sher-E-Kashmir University of Agricultural Sciences and Technology-Jammu, R.S Pura, Jammu, Jammu and Kashmir, India.

### Isolation and identification of bacteria

The nasal samples were inoculated on Columbia colistin nalidixic agar with 5% sheep blood agar and sheep blood agar and incubated aerobically at 37°C for 48 h for the isolation of Rhodococcal isolates. The bacterial isolates which showed small smooth shiny and nonhemolytic colonies after 24 h incubation but became larger, mucoid and salmon-pink in color with age were selected for further processing. Initial confirmation of the isolates as *R. equi* was done by demonstration of the typical cellular morphology in Gram-stained smears. All the nine isolates of *R. equi* obtained during the study were characterized biochemically by various standard biochemical tests, *viz*., catalase, oxidase, Christein-Atkin-Munch-Peterson (CAMP) test using *Staphylococcus aureus*, esculin hydrolysis, nitrate reduction test, urease test, and sugar fermentation test. The results of the biochemical tests for *R. equi* isolates are shown in Table-1.

**Table-1:** Biochemical tests for characterization of *R. equi.*

Test	Positive	Negative
Catalase test	9	0
Oxidase test	0	9
CAMP test	3	6
Esculin hydrolysis	0	9
Nitrate reduction test	9	0
Urease test	9	0
Glucose	0	9
Maltose	0	9
Sucrose	0	9

CAMP=Christein-Atkin-Munch-Peterson

### Antibiogram

All the isolates of *R. equi* were subjected to antibiotic sensitivity test by disc diffusion method using five antimicrobials amoxicillin - 30 mcg, penicillin G - 25 units, amikacin – 30 mcg, streptomycin - 10 mcg, and methicillin - 5 mcg [7].

### Molecular identification of isolates by polymerase chain reaction (PCR)

#### DNA extraction

The DNA template was extracted from purified bacterial colonies using snap and chill method. The DNA samples were further stored at −20°C till further use.

Species-specific *16S rRNA* gene was amplified by PCR with slight modifications [8]. PCR was carried out in a final reaction volume of 25 µl using 0.2 ml thin wall sterile and nuclease free PCR tubes.

#### PCR amplification

The PCR mixture contained a final concentration of 3.50 mM MgCl_2_, 0.2 mM concentrations of each deoxyribonucleoside triphosphates (dNTPs), 3.0 µl of ×10 PCR buffer, 1.0 μΜ of forward and reverse primers, 3.0 μl template DNA, and 1.0 U of taq DNA Polymerase (Promega limited, USA). The amplification cycle consisted of initial denaturation at 94°C for 5 min, followed by 35 cycles, each consisting of initial denaturation at 94°C for 30 s, annealing at 52°C for 30 s and extension at 72°C for 1 min which was followed by final extension at 72°C for 10 min. The sequence of forward and reverse primers used in the study is given in Table-2 with the predicted size of the PCR amplicon.

**Table-2:** List of oligonucleotide primers for detection of species specific *16S rRNA* gene.

Primer name	Nucleotide sequence	Product size (bp)
Forward primer	5’- GGTCTAATACCGGATATGAGCTCCTGTC 3’	450
Reverse primer	5’- CGCAAGCTTGGGGTTGAGCCCAA 3’	

### PCR-based detection of *R. equi* using Vap A gene

*Vap A* gene was amplified by PCR with slight modifications. PCR was carried out in a final reaction volume of 25 µl using 0.2 ml with final concentration of 3.50 mM MgCl_2_, 0.2 mM concentrations of each dNTPs and 2.5 µl of ×10 PCR buffer [9]. The amplification cycle consisted of initial denaturation at 94°C for 2 min, followed by 40 cycles, each consisting of initial denaturation at 94°C for 1.5 min, annealing at 57°C for 1 min and extension at 72°C for 2 min which was followed by final extension at 72°C for 10 min. Primer sequences used in the study and predicted size of the PCR amplicon is presented in Table-3.

**Table-3:** List of oligonucleotide primers for *Vap A* gene.

Primer name	Nucleotide sequence	Product size (bp)
Forward primer	5’- GACTCTTCACAAGACGGT-3’	563
Reverse primer	5’- TAGGCGTTGTGCCAGCTA-3’	

### Results and Discussion

**Figure-1:**
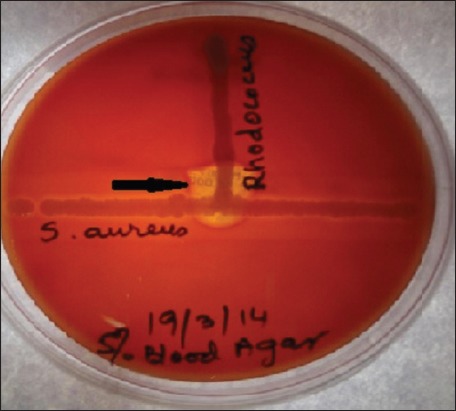
*Rhodococcus equi* showing spade shaped hemolysis on blood agar.

In this study, a total of nine isolates were recovered as *R. equi* on the basis of morphology, colony characteristics, and biochemical properties as described earlier. Six out of 9 samples were found positive for CAMP test as shown in Table-1. CAMP test shown as spade shape hemolysis on blood agar (Figure-1).

**Figure-2:**
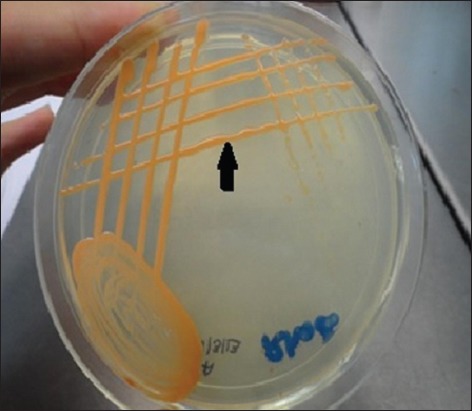
Salmon pink colored colonies of *Rhodoccus equi* on nutrient agar.

**Figure-3:**
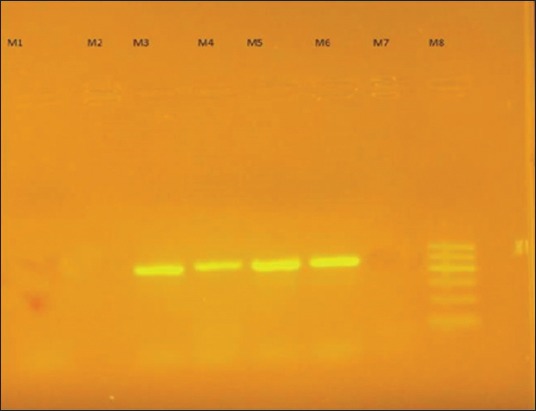
Amplified product of species specific *16S rRNA* polymerase chain reaction (PCR) of *Rhodococcus equi* at 450 bp. Lane M1, M2, M7 – Negative sample, Lane M3, M4, M5 – Amplified product of *16S rRNA* PCR of *R. equi* at 450 bp, Lane M6 – Positive control, Lane M8 – Molecular weight marker of 500 bp (Himedia, India).

The growth of *R. equi* on Columbia nalidixic agar with 5% sheep blood revealed small, smooth, shiny, and nonhemolytic colonies. After 24 h of incubation colonies became larger, mucoid and salmon – pink in color (Figure-2). The isolates of *R. equi* appeared as Gram-positive coccobacillary in Gram-stained smears. Out of nine, three *R. equi* isolates (two were from Katra and one from R.S. Pura [Table-4]) were confirmed positive as they revealed an amplicon of 450 bp (Figure-3) in 16S *rRNA* amplification. It had also been reported earlier that the detection rate of *R. equi* by *16S rRNA* PCR was 10.63% compared to 8.5% by culture [10,11]. However, it is recognized that PCR-based detection of *R. equi* from clinical samples is more sensitive than microbiological culture characterization [12]. PCR-based detection of *R. equi* is given in Table-4.

**Table-4:** *16S rRNA* gene showing result of PCR of *R. equi* from equines.

Region	Number of isolates subjected to PCR	Positive for *16S rRNA* gene
R.S. Pura	4	1
Katra	5	2
Total	9	3

*R. equi*=*Rhodococcus equi*, PCR=Polymerase chain reaction

In this study, the prevalence of *R. equi* recorded is less than that in an earlier report [13]. The low recovery rate of *R. equi* can be attributed to the fact that we did not use the invasive collection method of tracheobronchial aspiration which is regarded as the best method for definitive diagnosis of *R. equi* infection [14]. This method was not selected due to technical difficulty, reluctance by owners and associated life-threatening consequences. Another probable reason is the sporadic nature of the disease [15].

The virulence-associated antigens (Vap-A) and plasmids are used as epidemiological markers for *R. equi* virulence in foals [16]. In this study, none was found positive on PCR-based detection of *R. equi* using *Vap A* gene. However, observations were made, where a number of plasmid less *R. equi* isolates were found to be associated with clinical cases of foal pneumonia [17]. Therefore, it may be anticipated that occurrence of virulent plasmid may not entail the disease causing ability of *R. equi* rather it may intensify the pathogenic behavior of the organism and aggravate the disease prognosis. However, in one of the studies, it was found that environmental isolates are *Vap A* negative [18].

In the present study besides the recovery of target organisms, a variety of other bacterial isolates were obtained which were identified by conventional culture methods and by a set of standard biochemical tests. They were identified as *S. aureus, Pseudomonas*, *Proteus*, *Enterococcus*, and *Streptococcus pluranimalium*.

The antibiotic sensitivity assay for *R. equi* revealed that amoxicillin was the most effective followed by streptomycin and rifampicin. Results further revealed that resistance was highest for penicillin G. Results of antibiotic sensitivity assay for *R. equi* are presented in Table-5. Sensitivity patterns in most of the isolates, even within subspecies, varied remarkably. Earlier reports of the antimicrobial sensitivity on streptococcal isolates also indicated a wide variation in the sensitivity to various antibiotics used [19-21]. Although we did not find any multidrug-resistant strains, there is a need for the proper judicious use of antimicrobial agents for effective treatment and prevention of emergence of resistant strains. Resistance of the some of the isolates to a number of antibiotics seems to be outcome of indiscriminate use of those antibiotics in the field. The best therapeutic approach is a two-step approach. In the first step, combination of bactericidal drugs such as vancomycin plus imipenem is used to kill extracellular organisms and in the second step; a combination of drugs that penetrate the cell such as erythromycin plus rifampicin is administered for a period of at least 2 months [22]. The antibiogram study indicates that amoxicillin and streptomycin are the effective drugs used against bacterial pathogens. However, the emergence of drug resistance bacteria can be alarming which needs close and repeated vigilance. The indiscriminate use of antibiotics should be avoided. The different combination of antibiotics should be changed from time to time.

**Table-5:** Results of antibiogram for *R. equi*.

Antibiotic	Number of sensitive isolates	Number of resistant isolates	Number of intermediate isolates	% Sensitive	% Resistant	% Intermediate
Amoxicillin	3	0	0	100	0	0
Streptomycin	2	0	1	66.66	0	33.33
Penicillin	0	3	0	0	100	0
Methicillin	1	2	0	33.33	66.66	0
Rifampicin	2	0	1	66.66	0	33.33

*R. equi*=*Rhodococcus equi*

*R. equi* is an important pathogen which results in severe bronchopulmonary pneumonia not only in animals but also in humans. Every year, the state of Jammu and Kashmir is visited by thousands of tourists and pilgrims who use equines for recreational and religious purposes. During this time, they come in close contact with these equines and are at risk of getting infections from them and subsequently spreading them to other parts of the country. High infection rates of this pathogen have been associated with immunocompromised individuals, particularly AIDS patients [15].

## Conclusion

From this study, it was concluded that *R. equi* infection is prevalent in equine (Jammu region) in India, and as such, extensive studies with clinical samples from *R. equi*-infected and non-infected foals, adult equines, and environmental samples from various geographical locations are required for the molecular epidemiological analysis of *R. equi*. It was also found that indiscriminate use of the antibiotics is leading toward the development of resistant strains of *R. equi*.

## Authors’ Contributions

RJ and AKT designed the study. Laboratory work was done by RJ, GAB, RKS and AKT. All the authors participated in data analysis, while RJ drafted and revised the manuscript. All authors read and approved the final manuscript.
